# Higher-order optical rabi oscillations

**DOI:** 10.1016/j.fmre.2022.03.014

**Published:** 2022-04-03

**Authors:** Guohua Liu, Shenhe Fu, Siqi Zhu, Hao Yin, Zhen Li, Zhenqiang Chen

**Affiliations:** a Department of Optoelectronic Engineering, Jinan University, Guangzhou 510632, China; b Guangdong Provincial Key Laboratory of Optical Fiber Sensing and Communications, Guangzhou 510632, China; c Guangdong Provincial Engineering Research Center of Crystal and Laser Technology, Guangzhou 510632, China

**Keywords:** Rabi oscillations, Vector vortex light fields, Higher-order beams, Polarization control, Coupled mode system

## Abstract

Rabi oscillations express a phenomenon of periodic conversion between two wave states in a coupled system. The finding of Rabi oscillation has led to important applications in many different disciplines. Despite great progress, it is still unknown whether the Rabi oscillating state can be excited in the framework of the higher-order vector vortex regime. Here, we demonstrate in theory that the higher-order vector vortex light beams can be Rabi oscillating during evolution in an optical coupling system. This new classical oscillating state of light is characterized by a topologically shaped wavefront and coupled with spatially varying polarization. The vector vortex state exhibits a harmonic oscillatory property in the resonant and nonresonant conditions but differs greatly in Rabi oscillating frequencies. During Rabi oscillation, the complex state maintains its topology and intensity profile, while its intrinsic polarization pattern varies adiabatically in a periodic manner. We present an interpretation of the Rabi oscillation of the higher-order wave states in terms of the coupled-mode theory. Furthermore, we reveal a symmetry-protected transition between two Rabi oscillating modes, driven by a slowly varying phase mismatch. This Rabi transition has not been reported in either quantum mechanics or any other physical setting. This work advances the research of Rabi oscillation into the higher-order regime, and it may lead to novel applications in classical and quantum optics.

## 1 Introduction

The vector (polarization) state of a light beam is associated with its spin angular momentum and can be described as a point on the surface of the spin-based Poincaré sphere [1]. This geometric description relies on the orthogonal basis of two spin states represented by the north and south poles of the sphere [1], with any other state being a superposition of these two pure ones. When this spin basis is combined with the orbital angular momentum, it constructs a spin-orbit Poincaré sphere [2], [3], [4]. A combination of these spin-orbit bases produces higher-order vector vortex states characterized by a topologically shaped wavefront coupled to space-varying polarizations. Such higher-order light beams are represented by solutions to the Maxwell equations [5], [6] and can be generated by intracavity [7] or extracavity [8], [9], [10] techniques. Since the vector vortex beam displays intriguing correlations between the spin and the orbital angular momentum, studies of spin-orbit interactions of these structured light beams with matter have recently led to the discovery of impressive phenomena [11], [12], [13], [14], [15], [16], [17], including the conversion of the angular momentum between the spin and the orbit terms [13], [16] and the spin-orbit optical Hall effect [15]. Thus, vector vortex beams have drawn considerable interest, offering various applications in the context of classical and quantum optics alike [18], [19], [20], [21], [22]. The great potential shown by the vector vortex beams is complicated by difficulties in mapping their evolution onto the higher-order Poincaré sphere. A particular scenario allows the state to evolve adiabatically along a cyclic trajectory on the sphere, which resembles Rabi oscillations in two-level quantum systems [23], [24].

It is well known that Rabi oscillation was introduced by I. Rabi in 1937 as periodic population oscillations in a two-level quantum system driven by an external electromagnetic field [23]. Rabi oscillation gives rise to important applications, such as nuclear magnetic resonance (NMR) imaging and spectroscopy, and has become a cornerstone in many fields, including special quantum settings [25], semiconductors [26], atomic and molecular physics [27], [28], [29], [30], [31], acoustics [32], optics [33], [34], [35], [36] and photonics [37], [38], [39]. Notwithstanding its long history and progress to date, Rabi oscillation in light beams structured as complex vector vortices remains unclear.

In this article, we explore a dynamical structure that drives the motion of the vector vortex state on the higher-order Poincaré sphere and present a novel theoretical platform that allows the realization of the higher-order Rabi oscillation of light. We noted recently that the Rabi oscillation of a light state with plane wave polarization has been demonstrated in a microcavity [39]. However, this state of polarization is described by a spin-only-based Poincaré sphere; hence, it is considered a zero-order (scalar) wave state. We uncover, for the first time, that the higher-order vector vortex beams can be Rabi oscillating during evolution in an optical coupling setting, both in the resonant and the nonresonant conditions. Furthermore, we demonstrate via an adiabatic technique a symmetric-protected transition between two Rabi oscillatory modes that has been unnoticed in quantum mechanics or any other setting. We provide a physical platform that may implement the underlying higher-order Rabi oscillation of light.

## 2 Theory and results

### 2.1 Geometric theory for the vector vortex state

We start by considering a vector vortex state expressed as a superposition of two antipodal eigenstates [2]:

(1) $$\Phi =\left[\cos\left(\frac{\theta }{2}\right)\exp\left(-i\frac{\phi }{2}\right)\right]\hat{L}+\left[\sin\left(\frac{\theta }{2}\right)\exp\left(i\frac{\phi }{2}\right)\right]\hat{R}$$

where Φ is a normalized vector parameterized by the polar and azimuthal angles θ and ϕ, respectively, on the sphere, while eigenstates $\hat{R}=\exp\left(+il\varphi \right)\left(\hat{x}-i\hat{y}\right)/\sqrt{2}$ and $\hat{L}=\exp\left(-il\varphi \right)\left(\hat{x}+i\hat{y}\right)/\sqrt{2}$ represent two pure states with circular right and left polarizations, carrying opposite orbital angular momenta, l and −l, respectively. Here, $\hat{x}$ and $\hat{y}$ are unit vectors along axes x and y, respectively, and φ=arctan(y/x).

We explore a two-mode system in an optical birefringent material, in which the two vector vortex states exchange energy during state evolution. A linearly coupled mode theory can be utilized to analyze electromagnetic wave propagation and interaction between the two modes [40], [41], [42]. In the higher-order regime, the vector vortex state Φ is carried by a light field E(x,y,z), the full vector being $E=E\left(x,y,z\right)\Phi \equiv E_{x}\hat{x}+E_{y}\hat{y}$, where z is the propagation distance. The evolution of the vectorial light field in the coupling system is governed by coupled mode theory, which is written in a normalized form as

(2) $$\begin{array}{c} i\frac{\partial E_{x}}{\partial z}+\kappa E_{y}\exp\left(+i\Delta \beta \cdot z\right)=0\\ i\frac{\partial E_{y}}{\partial z}+\kappa E_{x}\exp\left(-i\Delta \beta \cdot z\right)=0 \end{array}$$

where κ denotes the mixing strength between $E_{x}$ and $E_{y}$, which is related to the refractive index distribution n(x,y) of the birefringent structure in the transverse plane. Δβ is the phase mismatch between the two components, i.e., the difference in the propagation constants of these two components. The parameters κ and Δβ play the roles of the coupling constant and energy difference, respectively, in a two-level quantum system, which supports Rabi oscillations [24]. Hence, the higher-order optical system can emulate the evolution of its quantum counterpart. Accordingly, the results reported below predict the higher-order Rabi oscillation in the two-component state with mixed vector vorticities of topologies +l and −l.

The solutions of Eq. 2 are tantamount to those for the Rabi oscillations in a two-level quantum system; hence, they can be mapped onto the respective Bloch sphere [24]. To realize geometric representation for the higher-order vector vortex states, we recall the Stokes parameters of light, which are real functions of the evolution axis and defined as [43]

(3) $$\begin{array}{ccc} S_{1}\left(z\right) & = & E_{x}E_{x}^{*}-E_{y}E_{y}^{*}\\ S_{2}\left(z\right) & = & E_{x}E_{y}^{*}+E_{y}E_{x}^{*}\\ S_{3}\left(z\right) & = & i(E_{x}E_{y}^{*}-E_{y}E_{x}^{*}) \end{array}$$

The Poincaré sphere for the higher-order light state is therefore constructed by these Stokes parameters. As a result, the Stokes vector $S\left(z\right)=[S_{1}\left(z\right),S_{2}\left(z\right),S_{3}\left(z\right)]$ at a given z can be mapped as a point onto the sphere. For instance, the north and south poles of the normalized sphere representing the two pure vortex states (see the corresponding states $\hat{R}$ and $\hat{L}$ in Eq. 1) can be represented by S=(0,0,1) and S=(0,0,−1), respectively. The motion equation of the Stokes vector S can be deduced according to Eqs. 2, 3, expressed in a Gyro form of

(4) $$\frac{dS}{dz}=g\times S$$

where × denotes the vector product. Here, $g=(g_{1},g_{2},g_{3})$ represents a torque vector directed to the vector vortex state. The three components of g are given by

(5) $$\begin{array}{ccc} g_{1} & = & 0\\ g_{2} & = & -2\kappa \cos(\Delta \beta \cdot z)\\ g_{3} & = & 2\kappa \sin(\Delta \beta \cdot z) \end{array}$$

These equations imply that g rotates with an angular rate Δβ, leading to a change in the vector vortex state. Therefore, the corresponding evolution trajectory of the complex light state can be mapped onto the surface of the higher-order Poincaré sphere.

To look for a solution for the higher-order Rabi oscillation, the explicit three-wave coupling equation for the Stokes vector S is given below:

(6) $$\begin{array}{ccc} \frac{dS_{1}}{dz} & = & -2\kappa \cos\left(\Delta \beta \cdot z\right)S_{3}-2\kappa \sin\left(\Delta \beta \cdot z\right)S_{2}\\ \frac{dS_{2}}{dz} & = & 2\kappa \sin\left(\Delta \beta \cdot z\right)S_{1}\\ \frac{dS_{3}}{dz} & = & 2\kappa \cos\left(\Delta \beta \cdot z\right)S_{1} \end{array}$$

Note that writing the lateral two equations of Eq. 6 as $2\kappa \sin\left(\Delta \beta \cdot z\right)=1/S_{1}\cdot dS_{2}/dz$ and $2\kappa \cos\left(\Delta \beta \cdot z\right)=1/S_{1}\cdot dS_{3}/dz$, and substituting these expressions for cos(Δβ·z) and sin(Δβ·z) in the first equation, casts it in the form of

(7) $$\frac{d}{dz}\left(S_{1}^{2}+S_{2}^{2}+S_{3}^{2}\right)=0$$

Hence, the system conserves the dynamical invariant, $S_{1}^{2}+S_{2}^{2}+S_{3}^{2}\equiv S_{0}^{2}=\mathrm{const}$, where $S_{0}={\left|E_{x}\right|}^{2}+{\left|E_{y}\right|}^{2}$. In a normalized form, one can set $S_{0}^{2}=1$. To obtain an analytical solution for the state vector, we assume that κ and Δβ are invariant with z. This assumption means that the refractive index distribution n(x,y) is independent of z. In this case, we define the dynamical variables:

(8) $$\begin{array}{c} S_{23}\equiv \sin\zeta \cdot S_{2}+\cos\zeta \cdot S_{3}\\ S_{32}\equiv \cos\zeta \cdot S_{2}-\sin\zeta \cdot S_{3} \end{array}$$

in terms of ζ≡Δβ·z. In this scenario, Eq. 6 can be expressed as an autonomous system:

(9) $$\begin{array}{ccc} \frac{dS_{1}}{d\zeta } & = & -2\kappa S_{23}/\Delta \beta \\ \frac{dS_{23}}{d\zeta } & = & 2\kappa S_{1}/\Delta \beta +S_{32}\\ \frac{dS_{32}}{d\zeta } & = & -S_{23} \end{array}$$

Exact solutions of the autonomous equations can be found:

(10) $$\begin{array}{ccc} S_{1} & = & \frac{2\kappa }{\Delta \beta \mu }\left[c_{1}\cos\left(\mu \cdot \zeta \right)-c_{2}\sin\left(\mu \cdot \zeta \right)\right]+c_{3}\\ S_{23} & = & c_{1}\sin\left(\mu \cdot \zeta \right)+c_{2}\cos\left(\mu \cdot \zeta \right)\\ S_{32} & = & \frac{1}{\mu }\left[c_{1}\cos\left(\mu \cdot \zeta \right)-c_{2}\sin\left(\mu \cdot \zeta \right)\right]+c_{4} \end{array}$$

where $\mu =\sqrt{4\kappa^{2}/\Delta \beta^{2}+1}$, and $c_{j}$ (j=1,2,3,4) is a constant that is related to the initial input. Therefore, the original variables $S_{2}$ and $S_{3}$ are expressed in terms of $S_{23}$ and $S_{32}$, leading to an analytic solution for S:

(11) $$\begin{array}{ccc} S_{1}\left(\zeta \right) & = & c_{3}+\frac{2\kappa }{\Delta \beta \mu }\left[c_{1}\cos\left(\mu \cdot \zeta \right)-c_{2}\sin\left(\mu \cdot \zeta \right)\right]\\ S_{2}\left(\zeta \right) & = & c_{4}\cos\left(\zeta \right)+\sin\left(\zeta \right)\left[c_{1}\sin\left(\mu \cdot \zeta \right)+c_{2}\cos\left(\mu \cdot \zeta \right)\right]\\ & & +\frac{\cos(\zeta )}{\mu }\left[c_{1}\cos\left(\mu \cdot \zeta \right)-c_{2}\sin\left(\mu \cdot \zeta \right)\right]\\ S_{3}\left(\zeta \right) & = & -c_{4}\sin\left(\zeta \right)+\cos\left(\zeta \right)\left[c_{1}\sin\left(\mu \cdot \zeta \right)+c_{2}\cos\left(\mu \cdot \zeta \right)\right]\\ & & -\frac{\sin(\zeta )}{\mu }\left[c_{1}\cos\left(\mu \cdot \zeta \right)-c_{2}\sin\left(\mu \cdot \zeta \right)\right] \end{array}$$

Considering the initial state $S\left(0\right)=[S_{1}\left(0\right),S_{2}\left(0\right),S_{3}\left(0\right)]$, the constant $c_{j}$ is obtained, expressed as

(12) $$\begin{array}{ccc} c_{1} & = & 2\kappa S_{1}\left(0\right)/\left(\Delta \beta \mu \right)+S_{2}\left(0\right)/\mu \\ c_{2} & = & S_{3}\left(0\right)\\ c_{3} & = & S_{1}\left(0\right)/\mu^{2}-2\kappa S_{2}\left(0\right)/\left(\Delta \beta \mu^{2}\right)\\ c_{4} & = & 4\kappa^{2}S_{2}\left(0\right)/{\left(\Delta \beta \mu \right)}^{2}-2\kappa S_{1}\left(0\right)/\left(\Delta \beta \mu^{2}\right) \end{array}$$

Note that, in a normal form, $S_{1}\left(0\right)=\sin\left(\theta \right)\cos\left(\phi \right)$, $S_{2}\left(0\right)=\sin\left(\theta \right)\sin\left(\phi \right)$, and $S_{3}\left(0\right)=\cos\left(\theta \right)$.

### 2.2 Rabi oscillations

**Fig. 1:**
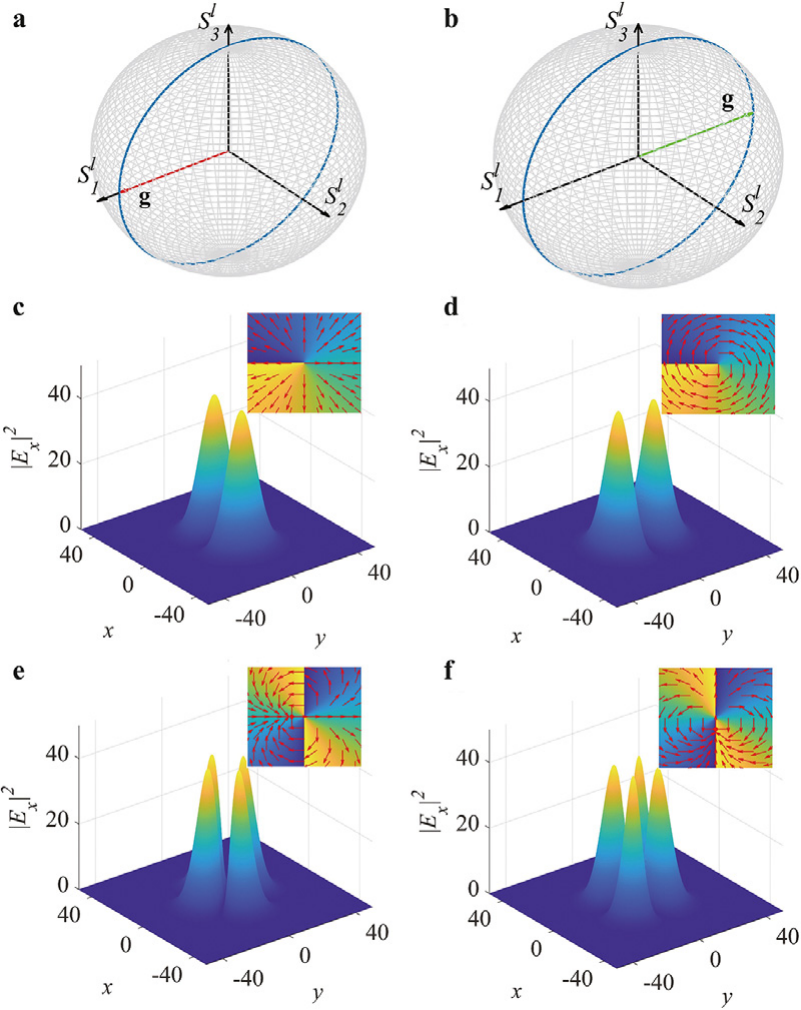
**Illustrations of the resonant higher-order Rabi oscillations**. (a,b) The vector vortex state starts from the position mapped on the Poincaré sphere with coordinates (θ,ϕ)=(π/2,0); see the torque vector g in (a); the torque vector rotates by angle π after a half-period evolution, z=π/(2κ) (here, κ=2). (b) The blue circle denotes the periodic evolution trajectory of the state on the sphere. (c,d) Corresponding intensity distributions of the x−component of the initial (c) and the converted (d) vector vortex beams with l=±1, produced by simulations of Eq. 2, initiated by the input in the form of Eq. 1. The insets show the respective phase patterns, with local arrows indicating the local polarization of light. (e,f) Same as in (c,d) but for l=±2.

First, we examine a situation with Δβ=0, i.e., perfectly matched components of the electromagnetic field, which is similar to a resonant two-level quantum system. In this case, Eq. 11 yields a particular harmonic solution for the Stokes vector:

(13) $$\begin{array}{ccc} S_{1}\left(z\right) & = & {\left[1-S_{2}^{2}\left(0\right)\right]}^{\frac{1}{2}}\cos\left(2\kappa z+\vartheta \right)\\ S_{2}\left(z\right) & = & S_{2}\left(0\right)\\ S_{3}\left(z\right) & = & {\left[1-S_{2}^{2}\left(0\right)\right]}^{\frac{1}{2}}\cos\left(2\kappa z-\vartheta^{\prime }\right) \end{array}$$

where the two offset angles in $S_{1}$ and $S_{3}$ are expressed as $\vartheta =\arctan[S_{3}\left(0\right)/S_{1}\left(0\right)]$ and $\vartheta^{\prime }=\arctan\left[S_{1}\left(0\right)/S_{3}\left(0\right)\right]$, respectively. The physical picture presented by the solution is as follows: when given an input of the vector vortex state that satisfies the ‘resonant’ condition, its $S_{2}$-component does not vary during state evolution on the sphere. Only the components of $S_{1}$ and $S_{3}$ accumulate a harmonic phase following a cosine oscillating function but with different offsets. Such cyclic evolution represents an intriguing Rabi oscillation with a Rabi frequency proportional to the coupling strength. To illustrate this picture, we start the process from a typical mixed state placed on the equator of the sphere, with angular coordinates expressed as (θ, ϕ)=(π/2, 0). The resultant initial state vector is S(0)=(1,0,0); see the torque vector g in Fig. 1a. After an evolution of half a cycle, z=π/(2κ), the torque vector g rotates by an angle of π, leading to a new Stokes vector S(z)=(−1,0,0), as shown in Fig. 1b. Therefore, the torque vector stimulates conversion between these two target states in the coupling setting. We present numerical observations for these complex state transitions. To this end, we simulated the real-time propagation of the vector vortex beams based on Eq. 2 by means of the fast Fourier transform algorithm. The beams carrying orbital angular momenta l=±1, see Eq. 1, are naturally shaped by the Laguerre-Gauss profile $E\left(r\right)=r\cdot \exp\left(-r^{2}/\sigma_{0}^{2}\right)$, where $r={\left(x^{2}+y^{2}\right)}^{1/2}$. The Gaussian width is chosen to be $\sigma_{0}=15$, for which the diffraction of light is negligible during propagation in the material. As shown in Fig. 1c and 1d, direct conversion among the vector vortex light states is clearly illustrated. Fig. 1c depicts the x-component of the initial state having space-varying radial polarization and coupled with the vortex phase (see the insert), whereas Fig. 1d shows its converted state (represented by its x-component) that maintains the vortex phase profile but features an azimuthal polarization pattern (see the insert). Note that these stimulated initial and target states agree with the mapping of the Stoke vector S in Fig. 1a and b, respectively. Thus, the vector vortex state oscillates during the evolution, featuring periodic changes in the cylindrically symmetric polarization patterns while keeping its topology and intensity profile unchanged. Our simulations suggest that the Rabi oscillation can be maintained in the presence of small perturbations, e.g., adding weak random noise to the initial state does not destroy the Rabi oscillatory light mode.

We further extend the periodic oscillating feature to even higher-order vector vortex states (l=±2); see the results in Fig. 1e,f. In this case, the spatially structured beam features doubled lobes in its x-component compared to the first-order light states. The light pattern in Fig. 1e exhibits a x−symmetry polarization distribution. After half an circle, the local polarization rotates by an angle of π/2 according to the torque vector g, giving rise to the y−symmetry polarization distribution; see the insert in Fig. 1f. Note that the increase in the topological charge of the light state does not alter the Rabi oscillating frequency.

**Fig. 2:**
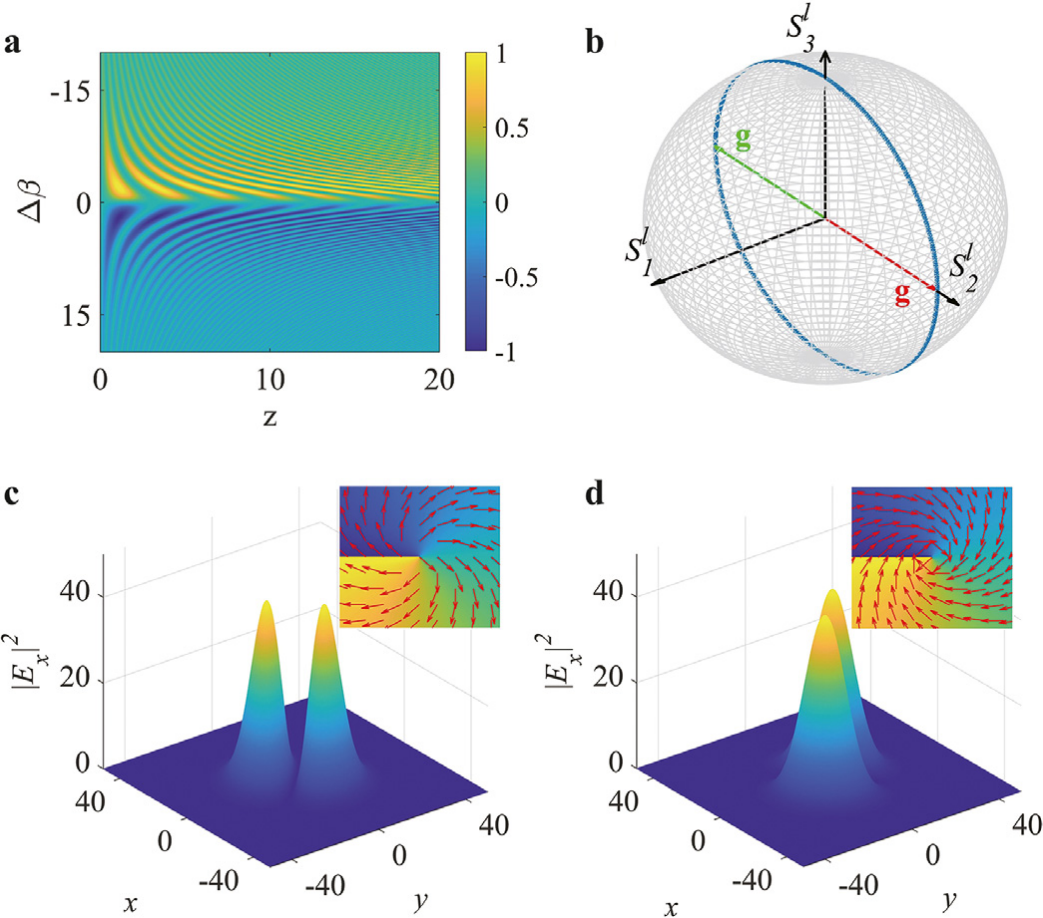
**Demonstration of the nonresonant higher-order Rabi oscillations**. (a) Value of $S_{1}$ as a function of Δβ and z for the input state: (θ, ϕ)=(π/2, π/2). (b) Illustration of a harmonic Rabi oscillatory mode in the nonresonant condition, with a Rabi frequency setting of $2\kappa^{2}/\Delta \beta =1/10$. (c,d) Simulated intensity distributions of the x−components of the initial (c) and the converted (d) vector vortex states, based on Eq. 2. The inserts in (c) and (d) display two vector vortex states with a topology of l=±1, corresponding to the mappings by the red and green torque vectors in (b), respectively. The local arrows in the inset indicate the local polarization of light.

While the stimulated periodic conversion of the vector vortex states is a resonant effect, we show that in the nonresonant condition, the vector vortex light beam also exhibits completely cyclic Rabi oscillations in the same coupling setting. Notably, the wave phenomenon of a complete Rabi oscillation in the detuned scenario has not been reported in either a two-level quantum system or any other physical setting, to our knowledge. In the present case of the higher-order regime, all components of the state vector rotate according to the Δβ−dependent torque vector. Fig. 2a presents the value of the Stokes component $S_{1}$ as a function of z and Δβ for the initial input state: (θ,ϕ)=(π/2, π/2). Apparently, any detuned Δβ results in noncyclic Rabi flopping, with relatively low oscillating amplitude, inversely proportional to μ·Δβ, as indicated by Eq. 11. The value of $S_{1}$ is π-phase shifted for opposite detuning. However, we reveal that with a sufficiently large phase mismatch, the imperfect Rabi flopping evolves into a perfect cyclic oscillatory mode. To observe this intriguing property, we set Δβ>κ. As a result, $\mu ≃1+2\kappa^{2}/\Delta \beta^{2}$, and Eq. 11 reduces to a harmonic oscillatory form:

(14) $$\begin{array}{ccc} S_{1}\left(z\right) & = & S_{1}\left(0\right)\\ S_{2}\left(z\right) & = & S_{2}\left(0\right)\cos\left(\frac{2\kappa^{2}}{\Delta \beta }\cdot z\right)-S_{3}\left(0\right)\sin\left(\frac{2\kappa^{2}}{\Delta \beta }\cdot z\right)\\ S_{3}\left(z\right) & = & S_{2}\left(0\right)\sin\left(\frac{2\kappa^{2}}{\Delta \beta }\cdot z\right)+S_{3}\left(0\right)\cos\left(\frac{2\kappa^{2}}{\Delta \beta }\cdot z\right) \end{array}$$

In this case, the nonresonant Rabi oscillating frequency is inverse to Δβ, suggesting a much slower oscillatory mode compared to the resonant case. Fig. 2b illustrates this dynamical state in terms of the first-order (l=1) Poincaré sphere, starting from the state vector (θ, ϕ)=(π/2, π/2), which corresponds to the red torque vector g. The corresponding light field and the polarization pattern (the insert) are depicted in Fig. 2c. After an evolution of $z=\pi \Delta \beta /(2\kappa^{2})$, the initial state gradually transforms into the opposite state, and the torque vector flips into its green counterpart, as displayed in Fig. 2b. Fig. 2d shows the corresponding converted light pattern, as well as its polarization distribution (the inset), generated by simulating the real-time evolution of light beams according to Eq. 2. Afterward, the reverse process begins, and the state gradually returns to its origin, exhibiting a detuned periodic Rabi oscillation of the higher-order vector vortex states.

### 2.3 Symmetry-protected Rabi transition

Finally, we reveal a symmetry-protected transition between two higher-order Rabi oscillating modes. Although the Rabi oscillations have been studied extensively in many physical systems [25], [26], [27], [28], [29], [30], [31], [32], [33], [34], [35], [36], [37], [38], [39], to our knowledge, this effect of the Rabi transition has never been reported. We demonstrate this property by modulating the phase mismatch profile along the evolution axis, exploiting the adiabatic technique [44]. Specifically, we set the phase mismatch quantity to be sufficiently large at the beginning (negative detuned) and the end (positive detuned) of the interaction process, which, as indicated in Fig. 2, will result in the respective nonresonant Rabi oscillating modes. We then tailor the sweeping rate of the phase mismatch along the axis so that it changes slowly with respect to the adiabatic condition [44]:

(15) $$|\kappa \cdot \frac{d\Delta \beta }{dz}|\ll {\left(\kappa^{2}+\Delta \beta^{2}\right)}^{3/2}$$

This behavior can be achieved by setting the phase mismatch profile following a nonlinearly chirped function: $\Delta \beta \left(z\right)=\Delta \beta_{0}\tanh\left(\gamma \cdot z\right)$, where $\Delta \beta_{0}$ is a constant offset, and γ is used to control the sweep rate. In this case, we note that the refractive index distribution is no longer independent of z but is subject to a corresponding modulation function of z.

**Fig. 3:**
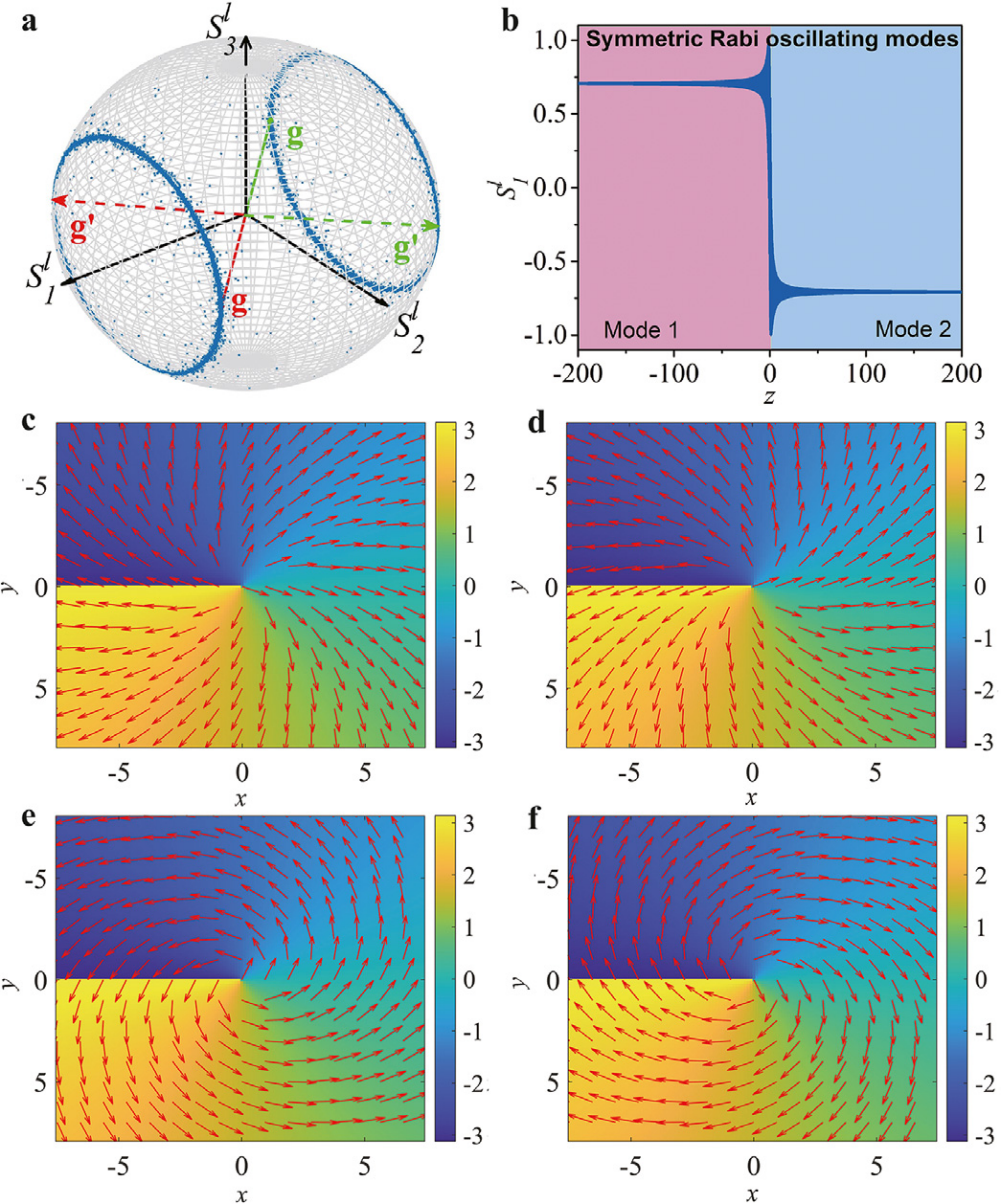
**Demonstration of the higher-order Rabi transition.** (a) Geometric illustration of the adiabatic Rabi transition between two oscillating modes for the input state: (θ, ϕ)=(π/2, π/4). The two red (green) torque vectors indicate the two oscillating states of the initial (final) Rabi mode. (b) z-dependence of the Stokes parameter $S_{1}$, indicating the transition from Rabi oscillatory mode 1 to mode 2. (c-f) Phase and polarization patterns in the initial (c,d) and final (e,f) Rabi modes, produced by simulations of Eq. 2. Panels (c,d) correspond to the mappings by the red solid- (c) and dashed-line (d) torque vectors, while (e,f) correspond to the green solid- (e) and dashed-line (f) torque vectors in (a).

We perform numerical analysis based on the three-wave coupling equation of Stokes **S** (Eq. 6), considering the typical settings: $\Delta \beta_{0}=1500$ and γ=0.01. Fig. 3a clearly identifies a transition from the initial oscillating mode (see the torque vector in red) to another mode (see the torque vector in green). The resultant oscillating mode is symmetric about the $S_{2}S_{3}$ plane and is a direct result of adiabatic energy transfer: under adiabatic conditions, the energy difference (equivalent to the population difference of two-level quantum states [24]) between the two orthotropic components of the light field should be maintained the same in these two oscillating modes, as illustrated in Fig. 3b. This figure shows a complete transfer of the energy difference from Rabi mode 1 to mode 2 within a short coupling length scale. We performed numerical observations based on Eq. 2 to confirm this phenomenon. Fig. 3c-f depicts the results of the Rabi oscillating modes before (Fig. 3c, d) and after the transition (Fig. 3e, f). We emphasize that the Rabi transition is symmetry protected, since any perturbation to the detuning, such as in the absence of an odd-symmetry profile, would not alter the final Rabi oscillating mode, yielding the same energy difference.

## 3 Discussion

We discuss possible physical settings that may implement the underlying higher-order optical Rabi oscillations. We note that the polarization distribution of a vector vortex wave state is not uniform in space, which makes it sensitive to the anisotropic structure. Therefore, a possible scheme for experimental realization is to exploit vector vortex light interactions with a structured anisotropic crystal, where the two state components undergo different refractive indexes, owing to the birefringent effect of the crystal. $n_{x}$, $n_{y}$ and $n_{z}$ represent the corresponding refractive index of the crystal along the coordinates x, y and z, respectively. Here, we have assumed that the vector vortex beam propagates along the z axis, which is in accordance with the optical axis. Paraxial coupled wave equations that describe the propagation dynamics of the vectorial light field in the crystal are given by [45]

(16) $$\begin{array}{c} i\beta_{x}\frac{\partial E_{x}}{\partial z}+\frac{n_{x}^{2}}{n_{z}^{2}}\frac{\partial^{2}E_{x}}{\partial x^{2}}+\frac{\partial^{2}E_{x}}{\partial y^{2}}=\gamma_{y}\frac{\partial^{2}E_{y}}{\partial y\partial x}\exp\left(+\frac{i}{2}\Delta \beta z\right)\\ i\beta_{y}\frac{\partial E_{y}}{\partial z}+\frac{n_{y}^{2}}{n_{z}^{2}}\frac{\partial^{2}E_{y}}{\partial y^{2}}+\frac{\partial^{2}E_{y}}{\partial x^{2}}=\gamma_{x}\frac{\partial^{2}E_{x}}{\partial x\partial y}\exp\left(-\frac{i}{2}\Delta \beta z\right) \end{array}$$

where $E_{x}$ and $E_{y}$ denote two Cartesian polarization components of the vectorial field that carries the vector vortex state Φ; see Eq. 1. $\beta_{j}=2k_{0}n_{j}$ (j=x,y) represents a propagation constant of the state component, where $k_{0}=2\pi /\lambda$ is the free-space wavenumber and λ is the carrier wavelength. $\Delta \beta =\beta_{y}-\beta_{x}$ is the phase mismatch quantity between the two orthogonal components. Hence, the terms exp(±iΔβz/2) demonstrate the accumulated phase difference between the two state components during propagation. This property is relevant to the difference in the refractive index of the crystal, i.e., $\Delta n=n_{y}-n_{x}$. $\gamma_{j}=1-n_{j}^{2}/n_{z}^{2}$ is an anisotropy parameter of the crystal that couples the two components $E_{x}$ and $E_{y}$. Clearly, this difference in the refractive index of the crystal gives rise to energy exchange, i.e., the coupling effect between the two components.

We assume that the crystal is not modulated in the transverse plane, and the incident vector vortex state is carried by a spatial mode that propagates through the crystal without changing the transverse mode profile of the two components. In this particular scenario, the diffraction terms (the second and third terms of Eq. 16) can be neglected. As a result, we simplify the coupled wave equation as

(17) $$\begin{array}{c} i\frac{\partial E_{x}}{\partial z}=C_{x}E_{y}\exp\left(+\frac{i}{2}\Delta \beta z\right)\\ i\frac{\partial E_{y}}{\partial z}=C_{y}E_{x}\exp\left(-\frac{i}{2}\Delta \beta z\right) \end{array}$$

where $C_{x}=\gamma_{y}/\beta_{x}\frac{\partial^{2}}{\partial y\partial x}$ and $C_{y}=\gamma_{x}/\beta_{y}\frac{\partial^{2}}{\partial x\partial y}$ represent the effective coupling among these two components. They are independent of the propagation distance. The reduced coupled wave equations (Eq. 17) are analogous to Eq. 2. Therefore, this specific system provides a platform to implement the predicted higher-order Rabi oscillation. Specifically, for a situation where $n_{x}=n_{y}$, the crystal supports an identical propagation constant of the two components, i.e., $\beta_{x}=\beta_{y}$, leading to a perfect phase match condition (i.e., Δβ=0). In this case, a phase-matched higher-order Rabi oscillation is expected. For a general situation $n_{x}\neq n_{y}$, the crystal gives rise to a detuned oscillatory mode, as mentioned above.

**Fig. 4:**
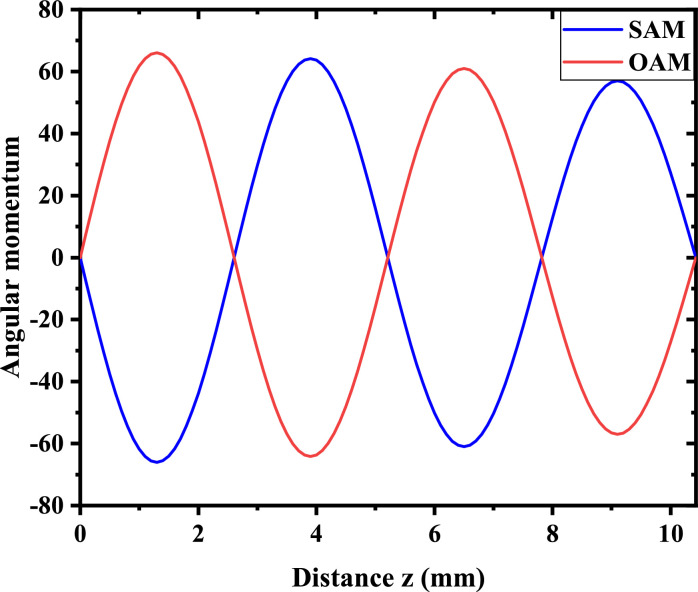
**Higher-order Rabi oscillation in a physical setting**. It shows spin-orbit angular momentum oscillation along the propagation distance in an anisotropic crystal, with $n_{x}=n_{y}=1.9929$ and $n_{z}=2.2154$. The vector vortex state is carried by a nondiffracting Bessel beam with a width setting of $r_{0}=2$μm. The state evolves from the equator (θ=π/2) of the first-order Poincaré sphere. The blue (red) curve denotes the variation in the spin (orbital) angular momentum of light.

To demonstrate the validity of the real model, we consider producing the ‘resonant’ higher-order Rabi oscillation. Since we have assumed that the input light beam should be nondiffracting, the initial Laguerre-Gauss profile is replaced by a first-order Bessel profile, $J_{1}\left(r/r_{0}\right)$ (here, $r_{0}$ determines the width of the Bessel profile), which exhibits a nearly nondiffracting property during propagation. We demonstrate the Rabi oscillatory phenomenon in terms of the optical angular momentum oscillation, as illustrated in Fig. 4. In the simulation, the size of the Bessel envelope carried by wavelength λ=633 nm is set as $r_{0}=2$ μm, and the crystal’s parameters are given by $n_{x}=n_{y}=1.9929$, $n_{z}=2.2154$. With these settings, our simulation suggests that $C_{x}\approx C_{y}$. The vector vortex state starts from the equator of the Poincaré sphere (θ=π/2), whose initial spin and orbital angular momentum (SAM and OAM) are zero, as indicated in Fig. 4. However, during state evolution in the crystal, the SAM and OAM exhibit approximately harmonic oscillation along the propagation distance, a direct result of the higher-order Rabi oscillation of light. Because of the conservation law, the separated SAM and OAM exhibit identical values but have opposite signs. Because of the limited calculation space, the Bessel function utilized in the simulation is imperfect, hence leading to a weak spreading of the envelope profile during propagation. This imperfect function causes a weakly damping amplitude of the angular momentum oscillation, as illustrated in Fig. 4.

## 4 Conclusion

We have shown a new form of Rabi oscillations in the framework of a spin-orbit-coupled optical Poincaré sphere, in which a higher-order vector vortex beam can be represented. We realized the new Rabi oscillating state via a two-mode coupling system, which might be implemented in an anisotropic crystal where the difference in refractive index couples the two state components. Notably, coupling between the two vector components may also be implemented in optical cavities [39], [46]. We presented a geometric theory to interpret these oscillatory modes in the phase matching and mismatching conditions. We found that when adiabatically modulating the phase mismatch profile, an effect of a symmetry-protected Rabi transition can be observed. Notably, although the Rabi oscillation is a very popular research topic [25], [26], [27], [28], [29], [30], [31], [32], [33], [34], [35], [36], [37], [38], [39], our result is the first prediction for the higher-order Rabi oscillations in the resonant and the nonresonant conditions.

We anticipate that the presented results apply to waves in spin-orbit-coupled two-component Bose-Einstein condensates (BEC) since wave propagation dynamics in the BEC setting are analogous to optical waves in many aspects, e.g., see the appearance of a vorticity-carrying state with higher-order topologies in [47], [48]. Because of the similarity of wave phenomena, it will also motivate intriguing investigations of spin-orbit-coupled wave phenomena in nonlinear optics [49], [50], [51] and electronics [52], [53]. In addition to these fundamental extensions, the finding of the Rabi transition between two oscillatory modes might find practical applications in optical switching, which requires designing a structure that implements a tailored phase mismatch profile along the propagation distance.

## Declaration of competing interest

The authors declare that they have no conflicts of interest in this work.

## References

[1] Born M., Wolf E. (1980).

[2] Milione G., Sztul H.I., Nolan D.A. (2011). Higher-order poincaré sphere, stokes parameters, and the angular momentum of light. Phys. Rev. Lett..

[3] Milione G., Evans S., Nolan D.A. (2012). Higher order pancharatnam-berry phase and the angular momentum of light. Phys. Rev. Lett..

[4] Yi X., Liu Y., Ling X. (2015). Hybrid-order poincaré sphere. Phys. Rev. A.

[5] Hall D.G. (1996). Vector-beam solutions of maxwell’s wave equation. Opt. Lett..

[6] Zhan Q. (2009). Cylindrical vector beams: From mathmatical concepts to applications. Adv. Opt. Photonics.

[7] Naidoo D., Roux F.S., Dudley A. (2016). Controlled generation of higher-order poincaré sphere beams from a laser. Nat. Photonics.

[8] Wang X., Ding J., Ni W. (2007). Generation of arbitrary vector beams with a spatial light modulator and a common path interferometric arrangement. Opt. Lett..

[9] Chen S., Zhou X., Liu Y. (2014). Generation of arbitrary cylindrical vector beams on the higher order poincaré sphere. Opt. Lett..

[10] Giordani T., Suprano A., Polino E. (2020). Machine learing-based classification of vector vortex beams. Phys. Rev. Lett..

[11] Bouchard F., Larocque H., Yao A.M. (2016). Polarization shaping for control of nonlinear propagation. Phys. Rev. Lett..

[12] Mueller J.P.B., Rubin N.A., Devlin R.C. (2017). Metasurface polarization optics: Independent phase control of arbitrary orthogonal states of polarization. Phys. Rev. Lett..

[13] Devlin R.C., Ambrosio A., Rubin N.A. (2017). Arbitrary spin-to-orbital angular momentum converison of light. Science.

[14] Guo C., Fu S., Lin H. (2018). Dynamic control of cylindrical vector beams via anisotropy. Opt. Exp..

[15] Fu S., Guo C., Liu G. (2019). Spin-orbit optical hall effect. Phys. Rev. Lett..

[16] Du L., Yang A., Zayats A.V. (2019). Deep-sub-wavelength features of photonic skyrmions in a confined electromagnetic field with orbital angular momentum. Nat. Phys..

[17] Yu P., Liu Y., Wang Z. (2020). Interplay between spin and orbital angular momenta in tightly focused higher-order poincaré sphere beams. Ann. Phys..

[18] Donato M.G., Vasi S., Sayed R. (2012). Optical trapping of nanotubes with cylindrical vector beams. Opt. Lett..

[19] Xie X., Chen Y., Yang K. (2014). Harnessing the point-spread function for high-resolution far-field optical microscopy. Phys. Rev. Lett..

[20] Liu J., Li S., Zhu L. (2018). Direct fiber vector eigenmode multiplexing transmission seeded by integrated optical vortex emitters. Light: Sci. Appl..

[21] Cozzolino D., Polino E., Valeri M. (2019). Air-core fiber distribution of hybrid vector vortex-polarization entangled states. Adv. Opt. Photonics.

[22] Sit A., Bouchard F., Fickler R. (2017). High-dimensional intracity quantum cryptography with structured photons. Optica.

[23] Rabi I.I. (1937). Space quantization in a gyrating magnetic field. Phys. Rev..

[24] Allen L.D., Eberly J.H. (1975).

[25] Chang B.Y., Sola I.R., Malinovsky V.S. (2018). Anomalous rabi osicllations in multilevel quantum systems. Phys. Rev. Lett..

[26] Schülzgen A., Binder R., Donovan M.E. (1999). Direct observation of excitonic rabi oscillations in semiconductors. Phys. Rev. Lett..

[27] Bludov Y.V., Konotop V.V., Salerno M. (2009). Rabi oscillations of matter-wave solitons in optical lattices. Phys. Rev. A.

[28] Mazzarella G., Malomed B., Salasnich L. (2011). Rabi-josephson oscillations and self-trapped dynamics in atomic junctions with two bosonic species. J. Phys. B: At. Mol.Opt. Phys..

[29] Dudin Y.O., Li L., Bariani F. (2012). Observation of coherent many-body rabi oscillations. Nat. Phys..

[30] Assemat F., Grosso D., Signoles A. (2019). Quantum rabi osicllation in coherent and in mesoscopic cat field states. Phys. Rev. Lett..

[31] Matthews M.R., Anderson B.P., Haljan P.C. (1999). Watching a superfluid untwist itself: Recurrence of rabi oscillations in a bose-einstein condensate. Phys. Rev. Lett..

[32] Cronenberg G., Brax P., Filter H. (2018). Acoustic rabi oscillations between gravitational quantum states and impact on symmetron. Nat. Phys..

[33] Kartashov Y.V., Vysloukh V.A., Torner L. (2007). Resonant mode oscillations in modulated waveguiding structures. Phys. Rev. Lett..

[34] Makris K.G., Christodoulides D.N., Peleg O. (2008). Optical transitions and rabi oscillations in waveguide arrays. Opt. Exp..

[35] Shandarova K., Rüter C.E., Kip D. (2009). Experimental observation of rabi oscillations in photonic lattices. Phys. Rev. Lett..

[36] Zhang P., Kang Q., Pei Y. (2020). Unveiling chiral phase evolution in rabi osicllations from a photonic setting. Phys. Rev. Lett..

[37] Dominici L., Colas D., Donati S. (2014). Ultrafast control and rabi oscillations of polaritons. Phys. Rev. Lett..

[38] Bose R., Cai T., Choudhury K.R. (2014). All-optical coherent control of vacuum rabi oscillations. Nat. Photonics.

[39] Colas D., Dominici L., Donati S. (2015). Polarization shaping of poincaré beams by polariton oscillations. Light: Sci. Appl..

[40] Shu W., Ling X., Fu X. (2017). Polarization evolution of vector beams generated by q-plates. Photonics Res..

[41] Xu Y., Chen S., Wang Z. (2019). Cylindrical vector beam fiber laser with a symmetric two-mode fiber coupler. Photon. Res..

[42] Wong G.K.L., Kang M.S., Lee H.W. (2012). Excitation of orbital angular momentum resonances in helically twisted photonic crystal fiber. Science.

[43] Goldstein D.H. (2011).

[44] Suchowski H., Porat G., Arie A. (2014). Adiabatic processes in frequency conversion. Laser Photonics Rev..

[45] Guo C., Fu S., Lin H. (2018). Dynamic control of cylindrical vector beams via anisotropy. Opt. Exp..

[46] Dufferwiel S., Li F., Cancellieri E. (2015). Spin textures of exciton-polaritons in a tunable microcavity with large TE-TM splitting. Phys. Rev. Lett..

[47] Kartashov Y.V., Zezyulin D.A. (2019). Stable multiring and rotating solitons in two-dimensional spin-orbit-coupled bose-einstein condensates with a radially periodic potential. Phys. Rev. Lett..

[48] Jin K., Li Y., Li F. (2020). Rabi oscillations of azimuthons in weakly nonlinear waveguides. Adv. Photonics.

[49] Karnieli A., Arie A. (2018). Fully controllable adiabatic geometric phase in nonlinear optics. Opt. Exp..

[50] Karnieli A., Trajtenberg-Mills S., Domenico G.D. (2019). Exprimental observation of the geometric phase in nonlinear frequency conversion. Optica.

[51] Karnieli A., Arie A. (2018). All-optical stern-gerlach effect. Phys. Rev. Lett..

[52] Karimi E., Marrucci L., Grillo V. (2012). Spin-to-orbital angular momentum conversion and spin-polarization filtering in electron beams. Phys. Rev. Lett..

[53] Verbeeck J., Tian H., Schattschneider P. (2010). Production and application of electron vortex beams. Nature (London).

